# A systematic review assessing the potential use of cystatin c as a biomarker for kidney disease in people living with HIV on antiretroviral therapy

**DOI:** 10.3389/fmed.2024.1295217

**Published:** 2024-03-19

**Authors:** Sidney Hanser, Joel Choshi, Haskly Mokoena, Sihle E. Mabhida, Zandile J. R. Mchiza, Marakiya T. Moetlediwa, Ndivhuwo Muvhulawa, Bongani B. Nkambule, Duduzile Ndwandwe, Unati Nqebelele, André P. Kengne, Phiwayinkosi V. Dludla

**Affiliations:** ^1^Department of Physiology and Environmental Health, University of Limpopo, Sovenga, South Africa; ^2^Non-Communicable Diseases Research Unit, South African Medical Research Council, Cape Town, South Africa; ^3^School of Public Health, University of the Western Cape, Bellville, South Africa; ^4^Department of Biochemistry, North-West University, Mmabatho, South Africa; ^5^Cochrane South Africa, South African Medical Research Council, Cape Town, South Africa; ^6^School of Laboratory Medicine and Medical Sciences, University of KwaZulu-Natal, Durban, South Africa; ^7^Department of Medicine, University of Cape Town, Cape Town, South Africa; ^8^Department of Internal Medicine, University of the Witwatersrand, Johannesburg, South Africa; ^9^Department of Biochemistry and Microbiology, University of Zululand, KwaDlangezwa, South Africa

**Keywords:** HIV, highly active antiretroviral therapy, biomarker, cystatin C, kidney function, nephropathy

## Abstract

The introduction of antiretroviral therapy (ART) has significantly prolonged the lifespan of people living with human immunodeficiency virus (PLWH). However, the sustained use of this drug regimen has also been associated with a cluster of metabolic anomalies, including renal toxicity, which can lead to the development of kidney diseases. In this study, we reviewed studies examining kidney disease in PLWH sourced from electronic databases such as PubMed/MEDLINE, Scopus, and Google Scholar, as well as gray literature. The narrative synthesis of data from these clinical studies demonstrated that the serum levels of cystatin C remained unchanged or were not affected in PLWH on ART, while the creatinine-based glomerular filtration rate (GFR) fluctuated. In fact, some of the included studies showed that the creatinine-based GFR was increased in PLWH taking tenofovir disoproxil fumarate-containing ART, perhaps indicating that the use of both cystatin C- and creatinine-based GFRs is vital to monitor the development of kidney disease in PLWH. Clinical data summarized within this study indicate the potential detrimental effects of tenofovir-based ART regimens in causing renal tubular injury, while highlighting the possible beneficial effects of dolutegravir-based ART on improving the kidney function in PLWH. However, the summarized literature remains limited, while further clinical studies are required to provide insights into the potential use of cystatin C as a biomarker for kidney disease in PLWH.

## 1 Introduction

Chronic kidney disease is a progressive medical condition that affects approximately 10% of the global population, with over 800 million reported cases worldwide (1). The International Society of Nephrology Global Health Atlas survey for Africa has estimated the prevalence of this condition to be similarly high in South Africa, especially aggravated/exacerbated by socioeconomic inequalities (2). Older individuals, including people with other disease conditions such as diabetes mellitus, hypertension, and human immunodeficiency virus (HIV), present with the greatest burden of kidney disease (3). Prominent features of chronic kidney disease include accelerated fibrosis and a decline in the rate of glomerular filtration, which can progress to end-stage kidney diseases and is associated with new-onset kidney injury (4). End-stage kidney failure occurs when the kidneys are no longer able to meet day-to-day requirements, while acute kidney injury is considered an abrupt reduction in the kidney function, which incorporates both structural damage and functional loss (5). Chronic kidney disease has become even more prevalent among people living with HIV (PLWH) compared to the general population and is correlated with an increased risk of adverse outcomes, such as heart failure, diabetes mellitus, and cardiovascular disease (6–8). Because of the many developing consequences associated with the sustained use of antiretroviral therapy (ART) (9, 10), there has been an enhanced need to understand the status of kidney disease in PLWH (11–13).

There is no argument that the effective use of ART has certainly prolonged the lifespan of PLWH (14). However, disparities linked with treatment adherence, dose selection, and the presence of other chronic conditions could contribute to disruptions in the organ function (15), including kidney toxicity (6, 7). The common methods of assessing the renal function in PLWH include the serum creatinine-based estimated glomerular filtration rate (eGFR) or creatinine clearance and/or urinalysis of proteinuria, glucose, or phosphate excretion (16, 17). However, evidence points to the limitations of these methods, as they may not be sensitive enough to detect kidney toxicity in PLWH (18–20).

Currently, growing evidence underscores the importance of making use of other alternative markers, such as serum cystatin C, to estimate GFR or renal tubular injury in PLWH (21, 22). Human cystatin C is a low-molecular-weight cysteine-rich protein that is synthesized by almost all tissues of the body (23). Despite the substantiated evidence that efficient kidneys can regulate serum cystatin C concentration to meet body’s homeostatic requirements, there is growing evidence to suggest an inverse correlation between GFR and serum cystatin C (20, 22, 24). Most recent evidence also shows that cystatin C could effectively identify high-risk chronic kidney disease individuals that may not have been detected by creatinine and enhanced eGFR-based risk stratification (25). However, it has also been argued that the available literature does not encourage the use of cystatin C or cystatin C-based equations to estimate the GFR in PLWH (26). This is compounded by the lack of consideration for other hypotheses regarding the potential detrimental effects of certain ART regimens on the kidney function in PLWH (27, 28). Thus, this aspect indicates the necessity for further research to scrutinize the relevance of using cystatin C or its relation to other biomarkers of kidney injury, such as creatinine, in PLWH on ART. Therefore, this report aimed to understand whether serum levels of cystatin C may be potentially used as a plausible biomarker to assess the renal function through the GFR. The current study, therefore, makes use of a systematic approach to identify and discuss clinical studies that provide information on the potential use of cystatin C as a biomarker for kidney disease or a risk thereof in PLWH. The analysis and discussion extend to evaluating whether ART has a negative or positive impact on the circulating levels of this protein in PLWH.

## 2 Methodology

### 2.1 Literature search strategy

This study was conducted following the Preferred Reporting Items for Systematic Reviews and Meta-Analyses (PRISMA) guidelines (29), although a meta-analysis was not conducted for this particular study (Supplementary File 1). Briefly, a systematic literature search was conducted using major online databases, such as PubMed/MEDLINE, Scopus, and Google Scholar, to identify clinical studies reporting on any association between cystatin C and kidney disease in PLWH on HARRT. The search strategy was compiled using a combination of the following keywords or Medical Subject Headings (MeSH); “cystatin C,” “ART,” “glomerular filtration rate,” “creatinine,” and “HIV” including the most relevant synonyms (Supplementary File 2). All the retrieved references were reviewed for additional relevant studies. This literature search was run without limitation until December 2023, for an adequate update on the literature. While this protocol was not registered with PROSPERO (International Prospective Register for Systematic Reviews), we conducted thorough searches of this database and any other relevant registries to ensure that our study did not duplicate existing research.

### 2.2 Study selection by inclusion and exclusion criteria

**Figure 1:**
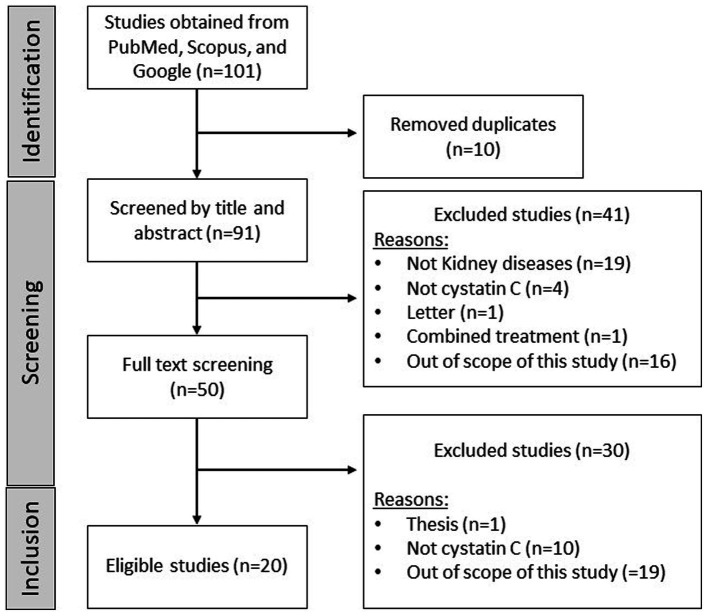
Flow diagram of the study selection process. Briefly, a systematic search of major electron databases retrieved 80 records, of which 81 were excluded for being duplicates or for not meeting the inclusion criteria. Notably, 20 studies (*n* = 20) were included and discussed within the manuscript, reporting on cystatin C and kidney disease in people living with HIV (PLWH) on antiretroviral therapy. Studies were mainly excluded because they were out of scope, one is a thesis or not reporting on the modulation of cystatin C.

Study selection was primarily conducted by two independent authors, focusing only on clinical articles meeting the criteria. Briefly, the inclusion criteria extend to articles that presented clinical research on kidney diseases, gray literature, pre-prints, and index journal publications, focusing on cystatin C and kidney disease in PLWH on ART. Exclusions were reviews, letters, editorials, notes, non-human studies, incomplete or unpublished studies, and those irrelevant to the scope (Figure 1). The following populations, interventions/exposures, comparators, outcomes (PICO/PECO) were used:

P: PLWH on ART
I/E: Levels of cystatin C (in PLWH on ART)
C: PLWH not on ART or treatment naive individuals
O: Markers/indicators of renal function.

### 2.3 Data extraction and quality assessment

Data extraction was independently conducted by two authors, with a primary focus on eligible studies reporting any association between cystatin C and kidney disease in PLWH on ART. The extracted items included author details, the year the study was published, the age of participants, the type of ART regimen, and the main findings reported in each report. Data reporting on other factors, such as ethnicity and existing chronic comorbidities, were also extracted, if available. The quality of included studies was also independently assessed by two authors, using the Downs and Black Checklist (30, 31). The checklist comprises 27 questions and 4 domains, allowing studies to be assessed as either good or excellent (29, 30, 32–35); of these, 12 received a score of good (26, 28, 31, 36–44) and 3 were rated as fair (25, 27, 45). Supplementary File 3 shows that included studies had low reporting bias with a mean score of 10 out of a possible score of 11 (overall agreement 85.67%, kappa = 0.71), excellent external validity with a mean score of 3 out of 3 (overall agreement 39.13%, kappa = 0.13), good internal validity with a mean score of 5 out of 7 (overall agreement 65.22%, kappa = 0.34), and low risk selection bias with a mean score of 4 out of possible 6 (overall agreement 56.51%, kappa = 0.19).

## 3 Results

### 3.1 General characteristics and quality of included studies

Briefly, a systematic search yielded nine qualifying clinical studies, published between 2008 and 2023 (Table 1). In terms of regional distribution, China contributed two qualifying studies, while evidence from other countries was broadly spread; the majority of studies were from the United States (*n* = 5), followed by Germany (*n* = 2), Nigeria (*n* = 2), Italy (*n* = 2), and China (*n* = 2), while other countries presented with one report, including France, Indonesia, Japan, Serbia, Spain, Poland, and the United Kingdom (Table 1). The study design included four randomized controlled trials, nine observational studies, four cross-sectional studies, one case–control, and two retrospective designs (Table 1). The study population involved PLWH on ART, with a sample size ranging from as low as *n* = 15 to as high as *n* = 670. Most studies (*n* = 10) encompassed adults with mean ages ranging from 34 to 53 years, one study involved young adults with a mean age of 17 years, and one study reported on children with a mean age of 12 years (Table 1). The study duration ranged from as low as 12 weeks to a maximum of 8 years of ART monitoring. However, it was also clear that most studies (*n* = 4) had a duration of approximately 48 weeks (Table 1). The quality of the included studies (*n* = 20) was assessed using the Downs and Black Checklist. Briefly, most of the studies (*n* = 15) were rated as having good quality of evidence, with two studies classified as having excellent quality evidence, scoring 25 and 27 out of 28 total scores (32, 45). Three of the 20 included studies were rated as fair quality studies, with scores of 17, 18, and 19 out of 28 possible scores (39, 43, 50). Based on the different domains, overall, there was an excellent reporting bias as indicated by a mean score of 10 (9–11) out of 11 possible scores, with a slight rater agreement between the two independent reviewers as indicated by a Cohen’s Kappa value (K) of 0.020. Furthermore, the studies exhibited overall good external validity with a mean score of 3 (0–3) out of 3 total scores and a fair rater agreement value of K = 0.30, and they also demonstrated overall good internal validity with a mean score of 5 (4–6) out of 7 total scores and a moderate rater agreement value of K = 0.42. The majority of the studies (*n* = 17) had good power (≥90%), indicating that these studies had no type 2 error, with a median score of 0.85 (0–1) (Supplementary File 3).

**Table 1:** Overview of clinical evidence linking cystatin C and kidney disease in people living with human immunodeficiency virus (PLWH).

References	Country	Type of study	Study population, including age	Intervention, including ART regimen and treatment duration	Main findings
Jones et al. (32)	United States	Cross-sectional study	PLWH on ART (*n* = 250), with a mean age of 41 years	Received ART for at least 1 year	Approximately 2.4% of the participants showed lower eGFRscr compared to approximately 15% showing low estimated glomerular filtration rate from creatinine (eGFR_cystC_)
Mauss et al. (33)	Germany	Observational study	PLWH on ART (*n* = 92), with a mean age of 37 years	Received ART for 3 years	Cystatin C correlated with HIV RNA and CD4+ T-cell count, but was suppressed after initiation of ART
Falasca et al. (34, 35)	Italy	Observational study	PLWH on ART (*n* = 15) with higher cardiovascular disease risk, with a mean age of 51 years	Received different types of ART for ≥12 months	Raised plasma levels of cystatin C were consistent with increased concentrations of leptin, interleukin-6/-18, and hypoadiponectinemia
Inker et al. (36)	United States	Observational study	PLWH on ART (*n* = 200), with a mean age of 48 years	Received ART with or without tenofovir, within 6 months after confirmation of HIV status	Cystatin C equation was not more accurate than the creatinine equation. The creatinine–cystatin C equation was more accurate than the cystatin C equation
Overton et al. (37)	United States	Observational study	PLWH on ART (*n* = 670) with renal dysfunction, with a mean age of 41 years	Received ART-containing tenofovir or ritonavir or both	Totally, 40% of subjects had renal dysfunction; 3.3% had chronic kidney disease. Elevated cystatin C was present in 18% of subjects
Bhasin et al. (38)	United States	Observational study	PLWH on ART (*n* = 187), with a mean age of 49 years	Received ART-containing tenofovir or cobicistat	eGFR_CystC_ bias and accuracy were strongly associated with the use of ART, HIV RNA suppression, and percentages of activated CD4 or CD8 T-cells
Yoshino et al. (39)	Japan	Retrospective design	PLWH on ART (*n* = 18), with a mean age of 44 years	Received ART-containing dolutegravir for ≥12 months	While the level of eGFR_CystC_ was not changed, that of the estimated glomerular filtration rate from creatinine (eGFRscr) fluctuated
Dragović et al. (40)	Serbia	Cross-sectional study	PLWH on ART (*n* = 33) with metabolic syndrome, with a mean age of 46 years	Received ART for at least 6 months	There was a positive correlation of cystatin C and C-reactive protein in PLWH with metabolic syndrome, compared to those without the metabolic syndrome
Szymczak et al. (41)	Poland	Observational study	PLWH on ART (*n* = 119) without a history of kidney dysfunction, with a mean age of 40 years	Received tenofovir disoproxil fumarate, protease inhibitors or 3 years	Low current CD4+ cell count was consistent with raised levels of cystatin C level. Use of tenofovir or other potentially nephrotoxic antiretroviral drugs did not have any impact on urinary cystatin C levels
Casado et al. (24)	Spain	Observational study	PLWH (*n* = 288) on ART, with a mean age of 50 years	Received dual regimens that included (dolutegravir + rilpivirine, 92; dolutegravir + darunavir/cobicistat, 23; dolutegravir, 26; cobicistat, 19; control group, 128) for 48 weeks	eGFRscr was reduced in PLWH taking two transporter inhibitors. This was similar to those taking dolutegravir or cobicistat. Similarly, the evolution of proteinuria and tubular dysfunction was apparent in all the groups, albeit no significant changes in eGFR_CystC_
Rashbaum et al. (45)	United States	Randomized controlled trial	PLWH (*n* = 725) on ART, with a mean age of 34 years	Received single-tablet regimen of darunavir/cobicistat/emtricitabine/tenofovir vs. darunavir/cobicistat + emtricitabine/tenofovir disoproxil fumarate (control) alafenamide for 48 weeks	Estimated glomerular filtration rate from serum cystatin C (eGFR_CystC_) remained unchanged between treatment groups. This was consistent with bone mineral density
Hamzah et al. (42)	United Kingdom	Randomized controlled trial	PLWH (*n* = 31) with a history of tenofovir disoproxil fumarate-associated proximal renal tubulopathy on ART, with a mean age of 53 years	Received 1:1 to continue current antiretroviral therapy or initiate emtricitabine/ tenofovir alafenamide for 12 weeks	eGFR_CystC_ and eGFRscr were not affected, including other makers such as albuminuria, proteinuria, renal phosphate or urea handling, (fasting) urine osmolality, parathyroid hormone, and bone turnover markers
John et al. (43)	Nigeria	Randomized controlled trial	PLWH on ART (*n* = 200), with a mean age of 27 years	Received ART-containing tenofovir or ritonavir or both	There was a significant difference between the values of creatinine, cystatin c, and urea recorded in PLWH on treatment
Priscilla et al. (44)	Nigeria	Case–control study	PLWH (*n* = 100) on ART, with a mean age of 37 years	Received lamivudine, stavudine, and nevirapine and monitored for no longer than 5 years	Serum cystatin C levels were elevated in men, regardless of ART status. This was correlated with serum creatinine levels
Zhao et al. (46)	China	Cross-sectional study	PLWH (*n* = 172) on ART, with a mean age of 40 years	Received tenofovir disoproxil fumarate + lamivudine + efavirenz, tenofovir disoproxil fumarate + plus lamivudine + ritonavir-boosted lopinavir, tenofovir disoproxil fumarate + lamivudine plus dolutegravir, or elvitegravir, cobicistat, emtricitabine and tenofovir alafenamide fumarate, and monitored for 1 year	eGFRscr alone was higher than eGFR calculated by the combination of serum creatinine and cystatin C, while that of eGFR_CystC_ was lower than eGFR calculated from both markers. This indicates that eGFR calculated by the combination of serum creatinine and cystatin C is more accurate, than each marker alone
Hikasa et al. (47)	Japan	Observational study	PLWH (*n* = 63) on ART, with a mean age of 39 years	Switched the antiretroviral drug from tenofovir disoproxil fumarate to tenofovir alafenamide, and exposures were 1.6 and 1.5 years, respectively	Switching from tenofovir disoproxil fumarate to tenofovir alafenamide was an independent predictor of improved eGFR_CystC_ slope
Lu et al. (48)	China	Retrospective design	PLWH (*n* = 138), over the mean age of 36 years	Received dolutegravir + tenofovir disoproxil fumarate, dolutegravir only, or tenofovir disoproxil fumarate for 48 weeks at most	Serum creatinine was significantly elevated in PLWH receiving dolutegravir + tenofovir disoproxil fumarate. Serum cystatin C and eGFR_CystC_ in those receiving dolutegravir were not affected, while eGFRcr was significantly higher in those receiving dolutegravir
Monin et al. (49)	Germany	Randomized controlled trial	PLWH (*n* = 263) on ART, with a mean age of 17 years	Switched to dolutegravir + boosted darunavir (with ritonavir) or continued with two nucleoside reverse transcriptase inhibitors in combination with ritonavir-boosted darunavir once daily for 48 weeks	eGFRscr was reduced while serum levels of cystatin C were not affected. Meanwhile, the low-density lipoprotein fraction was improved
Mondesert et al. (23)	France	Observational study	PLWH on ART (*n* = 262) with kidney dysfunction	Received cobicistat +elvitegravir, ritonavir + protease inhibitor, dolutegravir, dolutegravir +rilpivirine, rilpivirine, raltegravir, bictegravir, and other antiretroviral drugs	Mean eGFR_cystC_ was higher than mean eGFRscr
Rostania et al. (50)	Indonesia	Cross-sectional study	PLWH (*n* = 60) on ART PLWH with a mean age of 12 years	Received a combination of tenofovir, stavudine, lamivudine, zidovudine, abacavir, nevirapine, efavirenz, lopinavir/ for 8 years, at least	The serum cystatin C levels were high and correlated with high viral load in PLWH on ART. However, CD4^+^ count had no association with the serum levels of cystatin C

## 4 Discussion

### 4.1 Clinical evidence on the potential use of cystatin C to monitor kidney disease in PLWH on ART

Approximately nine studies reported on the use of cystatin C, together with the creatinine-based GFR, to monitor the kidney function in PLWH on ART (Table 1). However, nearly half of the studies included in this study supported the use of estimated GFR (from creatinine) as the preferred biomarker to assess or monitor the kidney function in PLWH on ART (42, 45, 46, 49). Interestingly, in addition to using GFR-based creatinine to monitor kidney disease, most of these studies favored the use of additional biomarkers to assess the kidney function in PLWH (Table 1). These biomarkers include albuminuria, proteinuria, and renal phosphate or urea handling, although cystatin C was predominantly used (Table 1). In terms of reliability between both creatinine- and cystatin-based GFRs, it was evident that the reported information was inconsistent between the studied subjects. For example, in some studies, it was shown that while the creatinine-based GFR was fluctuating, the levels of cystatin C remained unchanged and could be reliably used to monitor the kidney function in PLWH (24, 49), while other studies indicated that using both creatinine- and cystatin C-based GFRs could even be more reliable than using either marker alone in monitoring the kidney function in PLWH (36, 43, 46). Nonetheless, other studies indicated that cystatin C use alone could also be effectively used to detect the kidney function or kidney disease in PLWH (45), even correlating cystatin C levels with high viral loads in some participants (50). In other studies (34, 35, 40), it was also evident that elevated levels of cystatin C were consistent with increased levels of pro-inflammatory markers such as C-reactive protein, leptin, and interleukin-6/−18, possibly indicating other underlying conditions such as metabolic disease, which could contribute to the progression of kidney dysfunction in PLWH. Interestingly, in recent years, the rapid growth in the prevalence of metabolic diseases that is accompanied by abnormally high levels of inflammation has significantly affected PLWH (51–54), possibly contributing to the high burden of disease in many developing and developed countries.

### 4.2 Impact of ART on serum levels of cystatin C and other biomarkers of kidney disease in PLWH

It is important to note that the initial use of the zidovudine drug, which was approved in 1987 by the United States Food and Drug Administration, was instrumental in initiating the effective management of PLWH (55). Currently, many developments have been made in terms of managing PLWH, especially through the use of ART to suppress or reduce the viral load (56). In fact, the published literature predominately reports on recent ART combinations, especially protease inhibitors or nucleoside reverse transcriptase inhibitors, which are potentially linked with the development of non-communicable diseases, including kidney disease-associated risk factors (57–59). Notably, the sustained use of a tenofovir disoproxil fumarate-containing ART regimen has been linked with the detrimental effects of oxidative stress and inflammation, driving pathological abnormalities in renal cells (60, 61). This could be observed independently through the fluctuations in cystatin C levels in PLWH on ART (41). However, it was evident that the effective use of ART and suppression of CD4+ cell count were associated with low cystatin C levels in some patients (32, 33, 41). Thus, it remains important to understand the relationship between serum cystatin C and eGFR to monitor the kidney function in PLWH on ART. In this study, evidence indicated that the creatinine-based GFR increased in PLWH taking tenofovir disoproxil fumarate-containing ART (42, 45), but this was not observed in other studies (46, 48). Moreover, switching to a dolutegravir-based ART regimen could improve the kidney function in PLWH, as measured using both creatinine- and cystatin C-based GFRs (Table 1).

### 4.3 Summary and future perspective

Kidney injury is common in PLWH and has been associated with an increased risk of morbidity and mortality (62). Therefore, it has become imperative to routinely monitor biomarkers of kidney disease in PLWH. This aspect affirms existing literature on the necessity for screening, early diagnosis, and prediction of kidney disease in PLWH, especially those from low- and middle-income countries (63, 64). Although serum creatinine is considered an indirect marker for the renal GFR, it lacks specificity to detect damage to kidney tissue, and its relatively delayed response to injury could hinder early detection of acute kidney injury (65). Furthermore, studies have reported that serum creatinine levels are elevated with increasing age, body weight, and grip strength in some PLWH (66). This information renders this biomarker potentially unreliable to efficiently monitor the kidney function or the development of kidney disease. Clinical evidence from the current review indicates that while the measurement of creatinine-based GFR can fluctuate, cystatin C is likely to remain consistent in PLWH on ART. The use of both creatinine- and cystatin C-based GRF remains important and is recommended for monitoring the kidney function in PLWH on ART. However, the costs associated with the routine use of cystatin C together with creatinine to monitor the kidney function should be considered. In terms of the type of ART regimen, the use of tenofovir-containing ART may be potentially detrimental by increasing creatinine-based GFR as an indicator of kidney dysfunction in PLWH, while that of dolutegravir-based ART could be less toxic to the kidney. Evidence already supports the beneficial or superior effects of dolutegravir in combination with nucleoside reverse transcriptase inhibitors for suppressing HIV in PLWH (67, 68). This aspect underscores the need for more evidence to confirm these effects, especially since the cystatin C-based GFR was not affected. Further research is still required to provide a clear description of the modulation of these biomarkers to describe the relationship between both acute and chronic kidney dysfunction in PLWH on ART. Importantly, additional clinical evidence is required to determine the impact of different ART regimens on kidney injury in PLWH.

## Data availability statement

The original contributions presented in the study are included in the article/Supplementary material, further inquiries can be directed to the corresponding author.

## Author contributions

SH: Conceptualization, Funding acquisition, Investigation, Writing – original draft, Writing – review & editing. JC: Conceptualization, Writing – original draft, Writing – review & editing. HM: Writing – review & editing. SM: Funding acquisition, Writing – review & editing. ZM: Writing – review & editing. MM: Writing – review & editing, Data curation. NM: Writing – review & editing, Data curation. BN: Writing – review & editing. DN: Writing – review & editing. UN: Writing – review & editing. AK: Writing – review & editing. PD: Conceptualization, Funding acquisition, Writing – original draft, Writing – review & editing, Methodology, Supervision.
