# Supplementary material for: A systematic review assessing the potential use of cystatin c as a biomarker for kidney disease in people living with HIV on antiretroviral therapy
Source: Front Med (Lausanne). 2024 Mar 19;11:1295217. doi: 10.3389/fmed.2024.1295217 (PMC10985183; doi:10.3389/fmed.2024.1295217)
Supplement: Supplementary file 2 [file Data_Sheet_2.docx]

**PubMed database search terms for systematic review**

**Cystatin C related terms**

"Cystatin C"[All Fields]

**Renal function related terms**

("RENAL FUNCTION"[Title/Abstract] OR "Chronic kidney disease"[Title/Abstract] OR "CKD"[Title/Abstract] OR "Chronic renal insufficiency"[Title/Abstract] OR "Chronic kidney failure"[Title/Abstract] OR "Chronic renal failure"[Title/Abstract] OR "Chronic kidney insufficiency"[Title/Abstract] OR "gfr 60ml min"[Title/Abstract] OR "renal insufficiency"[Title/Abstract] OR "kidney insufficienc*"[Title/Abstract] OR "renal insufficienc*"[Title/Abstract] OR "kidney function"[Title/Abstract] OR "renal impairment"[Title/Abstract] OR "kidney impairment"[Title/Abstract] OR "renal disease*"[Title/Abstract] OR "kidney disease*"[Title/Abstract] OR "renal dysfunction"[Title/Abstract] OR "kidney dysfunction"[Title/Abstract] OR "renal failure*"[Title/Abstract] OR "kidney failure*"[Title/Abstract] OR "End Stage Kidney"[Title/Abstract] OR "End Stage Renal"[Title/Abstract])

**Drugs**

(("ART"[Title/Abstract] OR "antiretroviral treatment"[Title/Abstract] OR "cART"[Title/Abstract] OR "Combination Antiretroviral"[Title/Abstract] OR "HAART"[Title/Abstract] OR "Highly Active Antiretroviral Therapy"[Title/Abstract] OR "combination antiretroviral therap*"[Title/Abstract] OR "ANTIRETROVIRAL THERAPY"[Title/Abstract])

**HIV**

("Human immunodeficiency virus"[Title/Abstract] OR "HIV"[All Fields] OR "AIDS"[Title/Abstract] OR "Acquired immune deficiency syndrome"[Title/Abstract] OR "aids virus"[Title/Abstract] OR "acquired immune deficiency syndrome*"[Title/Abstract])

**Complete search strategy**

"Cystatin C"[All Fields] AND (("ART"[Title/Abstract] OR "antiretroviral treatment"[Title/Abstract] OR "cART"[Title/Abstract] OR "Combination Antiretroviral"[Title/Abstract] OR "HAART"[Title/Abstract] OR "Highly Active Antiretroviral Therapy"[Title/Abstract] OR "combination antiretroviral therap*"[Title/Abstract] OR "ANTIRETROVIRAL THERAPY"[Title/Abstract]) AND ("Human immunodeficiency virus"[Title/Abstract] OR "HIV"[All Fields] OR "AIDS"[Title/Abstract] OR "Acquired immune deficiency syndrome"[Title/Abstract] OR "aids virus"[Title/Abstract] OR "acquired immune deficiency syndrome*"[Title/Abstract]) AND ("RENAL FUNCTION"[Title/Abstract] OR "Chronic kidney disease"[Title/Abstract] OR "CKD"[Title/Abstract] OR "Chronic renal insufficiency"[Title/Abstract] OR "Chronic kidney failure"[Title/Abstract] OR "Chronic renal failure"[Title/Abstract] OR "Chronic kidney insufficiency"[Title/Abstract] OR "gfr 60ml min"[Title/Abstract] OR "renal insufficiency"[Title/Abstract] OR "kidney insufficienc*"[Title/Abstract] OR "renal insufficienc*"[Title/Abstract] OR "kidney function"[Title/Abstract] OR "renal impairment"[Title/Abstract] OR "kidney impairment"[Title/Abstract] OR "renal disease*"[Title/Abstract] OR "kidney disease*"[Title/Abstract] OR "renal dysfunction"[Title/Abstract] OR "kidney dysfunction"[Title/Abstract] OR "renal failure*"[Title/Abstract] OR "kidney failure*"[Title/Abstract] OR "End Stage Kidney"[Title/Abstract] OR "End Stage Renal"[Title/Abstract]))
